# Influence of etamsylate on coagulation parameters in dogs

**DOI:** 10.3389/fvets.2026.1734418

**Published:** 2026-03-04

**Authors:** Leonie Wörz, René Dörfelt, Katrin Hartmann, Vera Geisen

**Affiliations:** LMU Small Animal Clinic, Ludwig Maximilian University of Munich, Munich, Germany

**Keywords:** canine, dicynone, fibrinogen, hemorrhage, hemostasis, platelets, thromboelastography, viscoelastic tests

## Abstract

**Introduction:**

The mechanism of etamsylate on capillary bleeding and its influence on hemostasis is not yet fully understood. This study aims to evaluate the effects of etamsylate on thromboelastographic parameters, platelet count, clotting times and fibrinogen, and its adverse events in dogs.

**Methods:**

Dogs included in this prospective, non-randomized, single-arm interventional study with comparative analysis between clinical groups were divided into dogs without coagulopathies undergoing procedures with high risk of bleeding (*n* = 10), dogs with bleeding due to trauma or coagulopathy (*n* = 10), and thrombocytopenic dogs with a platelet count <80 × 10^9^/l (*n* = 10). Dogs that had previously received drugs or fluids influencing hemostasis were excluded. Blood samples were collected for clotting times, platelet count, thromboelastography, and fibrinogen analysis before and 90 min after etamsylate administration (12.5 mg/kg IV).

**Results:**

Thromboelastographic parameters, platelet count, clotting time, and fibrinogen concentrations did not change after treatment with etamsylate in the entire study population and in subgroup analyses. No adverse events of etamsylate were observed.

**Discussion:**

In the present study, no effect of etamsylate on hemostasis in dogs could be detected using coagulation parameters. Further studies using methods that incorporate the function of the vascular endothelium are necessary.

## 1 Introduction

Capillary hemorrhage in dogs can lead to life-threatening consequences. This hemorrhage can be caused by local lesions, trauma or systemic hemorrhagic diathesis, such as factor deficiency, thrombocytopenia, thrombocytopathy, or vasculopathy. Particularly in cases in which bleeding cannot be controlled mechanically, hemostatic drugs are used both preventively and therapeutically to shorten the duration of hemorrhage and prevent significant blood loss (1, 2).

Etamsylate (diethyl-ammonium-2,5-dihydroxybenzenesulfonate) is a hemostatic drug that has been used in human and veterinary medicine since 1959. After experimental parenteral or oral administration to rabbits, the mean bleeding time decreased (3). Etamsylate is primarily believed to act mainly by modulating primary hemostasis. Its mechanisms of action include activation of platelets through inhibition of prostacyclin release (4–6), enhancing platelet aggregation and adhesion via upregulation P-selectin membrane expression (7), and increasing the availability of platelet factor 3 and 4 (8). It also reduces vascular permeability through a so far unknown mechanisms (9, 10) and promotes vasoconstriction by inhibiting of prostacyclin release (5). Additionally, etamsylate antagonizes *in vitro* the heparin-induced increase in activated partial thromboplastin time (aPTT) in canine blood and reduces the heparin-induced prolonged bleeding time *in vivo* in rats. This effect has been proposed to result from interference of the etamsylate sulfonate group with the heparin-binding domains (11), as dobesylate has been shown to counteract the biological effects of vascular endothelial growth factor isoforms that contains such heparin-binding domain (12). When applied topically, etamsylate significantly shortened bleeding time in rats (11). Systemically applied etamsylate alone did not alter bleeding time in rats and did not affect aPTT in dogs (11). Etamsylate does not affect fibrinogen levels, platelet counts, and clotting times in humans or pigs (8, 13, 14).

A clinical study on the effect of etamsylate in 19 dogs with bloody diarrhea of unknown etiology demonstrated a beneficial effect. Dogs that received etamsylate (12.5 mg/kg intravenously every eight hours for seven days) in addition to standard therapy (metronidazole, trimethoprim-sulfadiazine, ranitidine, and metamizole) had a shorter time to normal feces (6.4 days vs. 10.7 days), a shorter duration of bloody defecation (3.3 days vs. 5.5 days), and less protein loss compared to the placebo group (15).

Proving the clinical efficacy of etamsylate remains challenging. In earlier studies, the efficacy of etamsylate was mainly clinically determined based on reduced blood loss (16), a shortened bleeding time (8), or a decreased incidence of intraventricular hemorrhage (5). Blood loss during surgery was evaluated either subjectively (17) or objectively using weighing swabs (14), colorimetric analysis of swabs (18), or by measuring the hemoglobin content of swabs (19). However, these methods are challenging to compare and lack reproducibility (20). Viscoelastic tests, including thromboelastography, assess secondary and tertiary hemostasis, but could also provide insights into primary hemostasis, particularly platelet function, which is thought to be the main target of etamsylate. Therefore, thromboelastography might be a suitable tool to detect etamsylate-induced effects on platelet function, which is primarily expressed by the maximal amplitude (MA).

Until now, the effects of etamsylate on clotting times, platelet count, and fibrinogen levels in dogs have not been investigated. An *in vitro* study investigated the influence of etamsylate on thromboelastographic parameters of canine blood (21). Unexpectedly, the addition of etamsylate to blood samples of dogs without coagulopathies resulted in increased lysis rates after 30 and 60 min. In a subsequent part of the study, higher doses of etamsylate were added to heparinized canine blood, reversing the anticoagulant effect of heparin and leading to increases in MA and alpha angle. However, all changes remained mild, were only achieved with high doses of etamsylate, and were not considered clinically relevant (21).

Therefore, this study aimed to evaluate the effect of intravenously administered etamsylate on thromboelastographic parameters, platelet count, clotting times, and fibrinogen levels in dogs. Additionally, adverse events of etamsylate application were documented.

It was hypothesized that etamsylate would primarily affect parameters reflecting primary hemostasis, especially by significantly increasing MA.

## 2 Materials and methods

The study protocol was approved by the ethics committee of the Centre for Clinical Veterinary Medicine, Ludwig Maximilians University Munich, Germany (276-25-07-2021). Written informed consent was obtained from all dog owners prior to participation.

### 2.1 Patient selection

Client-owned dogs presenting due to bleeding or for procedures associated with high risks of bleeding were considered for inclusion. Eligible dogs had either disorders of primary or secondary hemostasis, defined as thrombocytopenia (<80 × 10^9^/l), prolonged clotting times, hypocoagulable states in thromboelastography, clinical signs of bleeding (e.g., trauma), or were undergoing procedures with high-risk of bleeding (e.g., liver biopsy, liver aspiration, rhinoscopy with nasal biopsy) without any evidence of systemic bleeding tendencies, as determined by clinical examination and standard coagulation testing. Dogs were excluded if they had received anticoagulants, antiplatelet drugs, or fibrinolysis inhibitors within two weeks; metamizole or non-steroidal anti-inflammatory drugs within 48 h; blood products within three hours; or intravenous fluids exceeding 10 mL/kg/h within one hour prior to enrollment or during the study period. In the group of dogs undergoing procedures with high risk of bleeding, dogs with significant comorbidities affecting coagulation (e.g., severe liver disease, active infection) were excluded.

Sample size was calculated *a priori* to detect a mean increase in MA of 30% with *α* = 0.05 and 80% power, using the paired-samples formula and an estimated SD of the paired differences of 40%, which yielded a required sample of 28 dogs. The assumed standard deviation of the paired differences (40%) was based on variability reported in previous veterinary thromboelastography studies in dogs (22), in which standard deviations for maximum amplitude of approximately 10–20 mm has been described relative to mean values of around 60 mm, and was conservatively increased to account for greater heterogeneity in a clinical population.

The following breeds were represented: mixed-breed dogs (*n* = 9), Golden Retriever (*n* = 2), French Bulldogs (*n* = 2), and one dog each of the following breeds: Rhodesian Ridgeback, Border Collie, German Shepherd, collie, rottweiler, dachshund, Old English Bulldog, poodle pointer, poodle, pug, maltese, German Shorthaired Pointer, Bavarian Mountain Hound, Jack Russell Terrier, labradoodle, toy poodle, and Swiss Shepherd. The median age was 7 years (range: 0.5–14.0), and the median body weight was 22 kg (range: 3–42). The study population comprised 18 female dogs (11 spayed) and 12 male dogs (8 neutered).

### 2.2 Study design

A prospective, non-randomized, single-arm interventional study with comparative analysis between clinical groups was performed. All included dogs were categorized into one of three groups: one group comprised ten dogs without suspected bleeding tendencies undergoing procedures with high risks of bleeding (liver biopsy, liver puncture, or rhinoscopy). Another group included ten dogs presenting with bleeding due to trauma or systemic bleeding diathesis. The third group consisted of ten dogs with thrombocytopenia (< 80 × 10^9^/l). The cutoff for the thrombocytopenia group was selected based on a study in dogs with immune-mediated thrombocytopenia, which demonstrated a normal MA on thromboelastography when platelet counts exceeded 80 × 10^9^/l (23). Before enrollment, a detailed medical history was obtained, particularly regarding the drugs administered in the previous two weeks, and a physical examination was performed, with specific attention to bleeding, petechiae, and hematomas.

Etamsylate 125 mg/mL (Hemosilate, Ecuphar, Spain) was administered intravenously at a dose of 12.5 mg/kg. Adverse events were monitored over a three-hour study period by repeated clinical evaluations (mucous membranes, heart rate, pulse quality, breathing rate, hematomas, petechia or bleeding) every 30 min. Blood samples were collected before and 90 min after administration of etamsylate. This time point was selected because the maximum effect following intravenous administration was determined to be reached at 60 min and to last for approximately four hours (24). Three milliliters of blood were collected free-flowing from a cephalic vein through an IV catheter immediately after insertion (before etamsylate administration) and from the lateral saphenous vein using a 20-G cannula (90 min after etamsylate administration). Blood was first drawn into two 3.2% sodium citrate tubes in a strict 1:9 ratio, followed by 1 ethylenediaminetetraacetic acid (EDTA) tube.

### 2.3 Laboratory measurements

Complete blood count (CBC) (Sysmex XT-2000i hematology analyzers; Sysmex Corporation, Kobe, Japan) (including manual platelet count, if the platelet count was below the reference range), prothrombin time (PT) and activated partial thromboplastin time (aPTT; CL 4 analyzer; Behnk Elektronik, Norderstedt, Germany), fibrinogen (Clauss fibrinogen assay; ANTECH Diagnostics, Augsburg, Germany) and thromboelastography (Haemoscope Thromboelastograph^®^ analyzer, model TEG^®^ 5,000; Haemonetics Corp, Boston, United States) were measured.

One citrated tube was directly centrifuged for analysis of the clotting times (ball coagulometer). For PT analysis, 50 μL of citrated plasma was diluted with 950 μL of imidazole buffer. Then, 100 μL of this diluted plasma was incubated at 37 °C with 100 μL of fibrinogen solution for two minutes. After adding 100 μL of neoplastin, the time to complete coagulation was measured. For aPTT analysis 100 μL of citrate plasma were mixed with 100 μL of partial prothrombin time reagent (C.K. Prest, Cromakit, Maracena, Andalusia, Spain). After a three-minute incubation, 100 μL calcium chloride was added, and the time to complete coagulation was recorded.

The remaining citrated plasma was chilled at 5–8 ° Celsius and shipped to an external laboratory (ANTECH Diagnostics, Augsburg, Germany) at this temperature. External laboratory performed the Clauss fibrinogen assay. In which the concentration of functional fibrinogen in the blood sample was measured by adding a known amount of thrombin and measuring the time until clot formation.

According to the PROVETS guidelines, the second tube of citrated blood was stored at room temperature for 30 min before processing it for thromboelastography (25). One mL sample was added to a kaolin cup for activation. Tubes were inverted five times and 20 μL of calcium chloride, followed by 340 μL of blood were manually pipetted into the prewarmed cups at 37 °C. The measurements were started immediately after the introduction of the pin into the blood (25). The measured variables included reaction time (R), *α* angle, k time (K), maximal amplitude (MA), and clot lysis after 30 min (LY30). The global clot strength (G) was subsequently calculated using the following formula: G = (5,000 × MA)/(100−MA) (26).

### 2.4 Statistical analysis

All patient values were compared before and after etamsylate administration. The comparisons were performed within the three main groups. Furthermore, separate analyses were conducted for subgroups based on platelet count, presence of active bleeding, and the MA value (dogs with decreased MA vs. those with MA within reference range). Based on this comparative statistical analysis of the subgroups with different MA values, the aim was to determine whether the effect of etamsylate is detectable only in patients with normal primary hemostasis (MA values within the reference range) or only in patients with impaired primary hemostasis (MA values below the reference range).

Statistical analyses were performed using a commercially available software package (GraphPad Prism, Boston, United States). Normality of data was assessed using D’Agostino-Pearson omnibus test. Normally distributed data are presented as mean ± standard deviation. Non-normally distributed data are presented as median and range (minimum–maximum). All pre- and post-administration values were compared using the Wilcoxon matched-pairs signed-rank test. To control for family-wise error rate due to multiple comparisons, a Holm–Bonferroni step-down procedure was applied. Accordingly, adjusted *p*-values were used, and *p*-values ≤ 0.05 after correction were considered statistically significant.

## 3 Results

A total of 30 client-owned dogs were included in the study. All enrolled patients completed the study. Eight of the enrolled dogs were euthanized and two dogs died after the study period due to the severity of their underlying disease.

### 3.1 Thromboelastography

**Figure 1:**
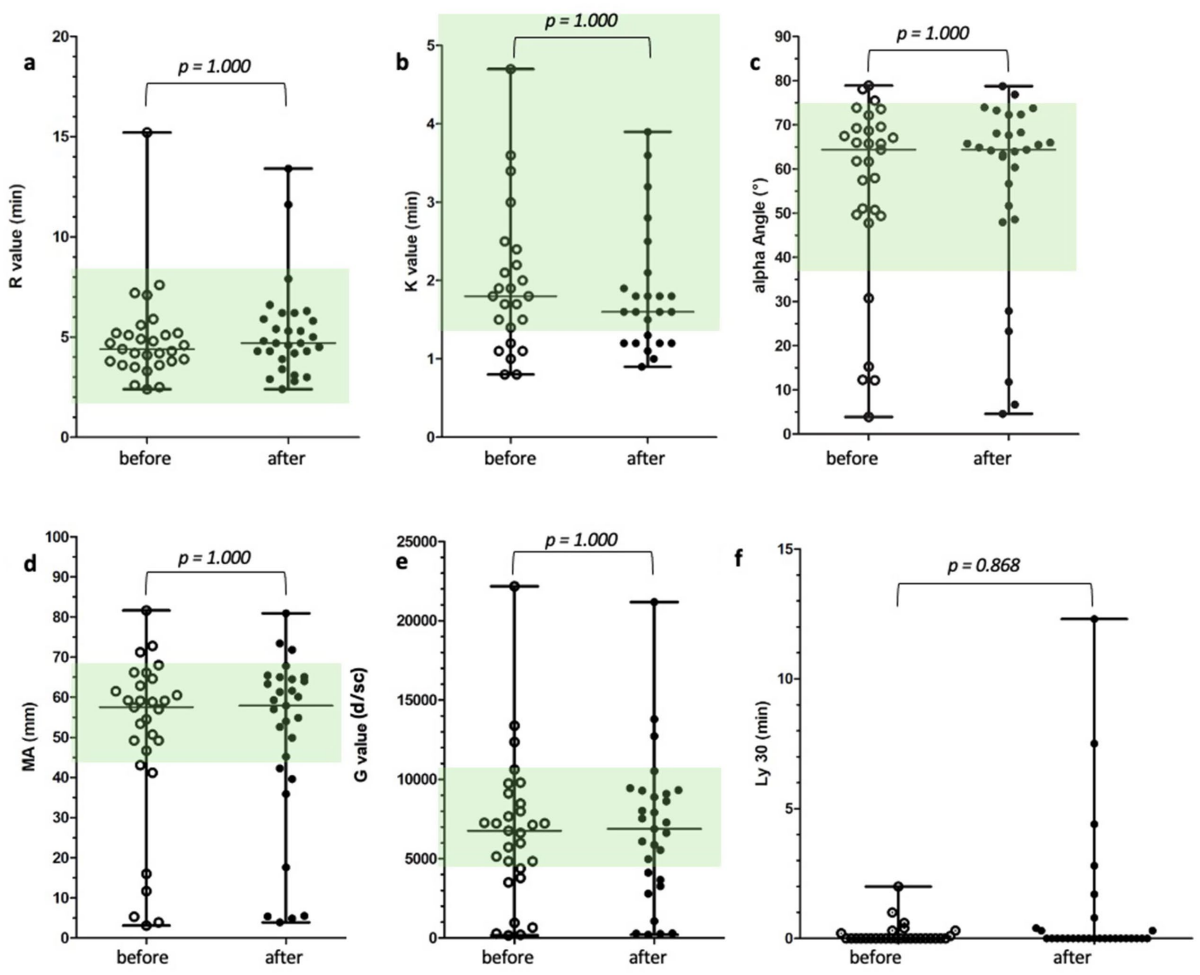
**(a–c)** Scatter dot plots of thrombelastographic parameters before and after treatment with etamsylate. Number of patients per parameter: R value (*n* = 30), K value (*n* = 24), alpha angle (*n* = 29). Wilcoxon matched-pairs signed-rank test showed no significant difference. The green colored areas represent the reference range. K = k time, R = reaction time. *p-*values ≤ 0.05 were considered significant. **(d–f)** Scatter dot plots of thrombelastographic parameters before and after treatment with etamsylate. Number of patients per parameter: MA (*n* = 29), G value (*n* = 29), LY30 (*n* = 29). Wilcoxon matched-pairs signed-rank test showed no significant difference. The green colored areas represent the reference range. G = global clot strength [G = (5,000 × MA)/(100−MA)], LY30 = clot lysis at 30 min, MA = maximal amplitude. *p-*values ≤ 0.05 were considered significant.

Thromboelastography was performed in all patients and results were analyzed in 24 patients, because in five patients with thrombocytopenia and one patient with a secondary coagulation disorder, the K time could not be determined because the required clot amplitude was not reached. In one dog, MA, LY30 and angle were also outside the measurable range. These patients were excluded from the statistical analysis of the affected parameters. No significant differences were observed in the thromboelastographic parameters between before and after etamsylate samples, both among all patients (Table 1; Figures 1a–f) and when analyzing the three individual groups.

**Table 1:** Coagulation times, platelet count, fibrinogen, and thromboelastography values of 30 dogs before and after etamsylate.

Parameters (*n*)	Before etamsylate		After etamsylate		Reference range	*p*-values
	Median	Range	Median	Range		
R (min) (*n* = 30)	4.4	2.4–15.2	4.7	2.4–13.4	1.8–8.6	0.173
K (min) (*n* = 24)	1.8	0.8–4.7	1.6	0.9–3.9	1.3–5.7	1.000
Angle (deg) (*n* = 29)	64.4	3.9–78.9	64.4	4.6–78.8	36.9–74.6	1.000
MA (mm) (*n* = 29)	57.5	3.1–81.6	57.9	3.9–80.6	42.9–67.9	1.000
LY30 (%) (*n* = 29)	0	0–2	0	0–12.3	0	0.868
G (d/sc) (*n* = 29)	6,765	160–22,174	6,877	203–21,178	3,757 – 10,576	1.000
Platelet count (× 10^9^/l) (*n* = 30)	198.5	1–442	178.5	2–391	148–484	0.120
Fibrinogen (mg/dl) (*n* = 22)	213	60–943	207	65.5–734	88–560	1.000
PT (sec) (*n* = 29)	19.2	13.5–41.4	20.3	12.3–45.8	13.8–23.2	0.216
aPTT (sec) (*n* = 29)	12.9	9.2–54.9	13.2	11.3–55.4	10–13.1	1.000

aPTT = activated partial thromboplastin time, G = global clot strength [G = (5,000 × MA)/(100−MA)], K = k time, LY30 = clot lysis at 30 min, MA = maximal amplitude, PT = Prothrombin time, R = reaction time. *p*-values ≤ 0.05 were considered significant.

In the subgroup analysis comparing patients with a platelet count below 80 × 10^9^/l and those with a platelet count above 80 × 10^9^/l as well as patients with hemorrhage and those receiving etamsylate due to high-risk bleeding procedures, no significant differences were observed in thromboelastographic parameters between pre- and post-treatment values.

**Figure 2:**
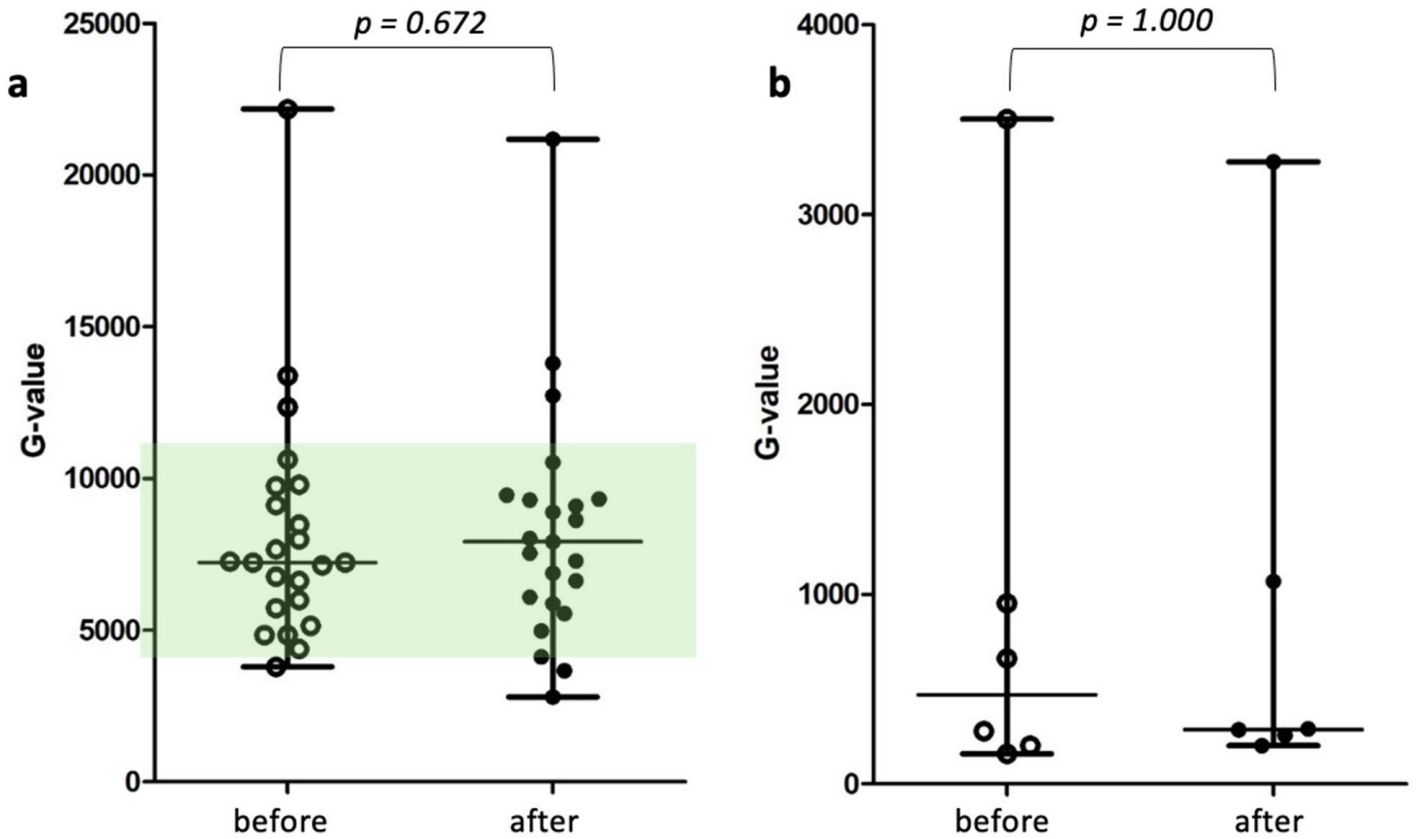
**(a,b)** Scatter dot plots of calculated global clot strength [formula: G = (5,000 × MA)/(100−MA)] before and after treatment in the subset of patients with normal MA **(a)** (*n* = 24) and the subset of patients with decreased MA **(b)** (*n* = 6). Wilcoxon matched-pairs signed-rank test showed no significant difference. The green colored area represents the reference range. Due to the lower G values in patients with decreased MA, the y-axis in **(b)** was adjusted accordingly. A green-colored area is not displayed, as all values fall markedly below the reference range. G-value = global clot strength, MA = maximal amplitude. *p-*values ≤ 0.05 were considered significant.

In the subgroup with decreased MA, no significant differences in thromboelastographic parameters were detected before and after etamsylate administration. The corresponding G values are shown in Figures 2a,b.

### 3.2 Platelet count

When analyzing all dogs, the median platelet counts decreased from 198 × 10^9^/l (range: 1–442 × 10^9^/l) to 179 × 10^9^/l (range: 2–391 × 10^9^/l) after administration of etamsylate (Table 1). These values were not significant after the Holm–Bonferroni step-down procedure (*p* = 0.120). In none of the dogs did the platelet count decrease below the predefined cut-off value of 80 × 10^9^/L after etamsylate administration if the platelet count had been within the normal range beforehand. Likewise, no significant decrease in platelet count was observed in the subgroup analyses [dogs with normal platelet count (*p =* 0.08), dogs with normal MA (*p =* 0.140), dogs that received etamsylate due to a risk of bleeding (*p =* 0.322)].

### 3.3 Clotting times

One dog was excluded from the clotting times analysis due to pre-analytical errors. In the remaining 29 dogs, no significant differences in aPTT (*p =* 1.000) and PT (*p =* 0.216) were observed between samples collected before and after etamsylate administration. Also, in the subgroup analysis no significant differences were observed in clotting times between pre- and post-treatment values (see Supplementary material).

### 3.4 Fibrinogen

Fibrinogen values were available for 22 of the 30 enrolled dogs. In eight dogs, measurement was not possible due to laboratory errors (e.g., insufficient blood sample volume, sample loss during shipment). No significant difference in fibrinogen levels was observed before and after etamsylate administration (*p* = 1.000; Table 1). In the subgroups of dogs with and without bleeding and those with or without thrombocytopenia, no significant differences before and after etamsylate administration were detected.

### 3.5 Adverse events

No adverse events related to etamsylate were observed during the study period.

## 4 Discussion

Intravenous administration of 12.5 mg/kg etamsylate to dogs did not result in significant changes in coagulation parameters and platelet count.

Since etamsylate is assumed to act mainly on primary hemostasis, TEG was considered a suitable method to assess its efficacy. The MA primarily reflects clot firmness, 80–90% of which is determined by platelet count and function. However, no effects on any of the thromboelastographic parameters were observed in this study. Additionally, subgroups of dogs with normal and low platelet counts were analyzed, but also in both of those subgroups no effects of etamsylate were detectable.

Although experimental studies have demonstrated an effect of etamsylate on various steps of primary hemostasis, its exact mechanism of action remains unclear. A procoagulant effect of etamsylate might not have been detectable in the present study due to only assess the *ex vivo* results, which did not include the effect of the vascular endothelium and vascular tone in the context of primary hemostasis. Furthermore, although etamsylate might be effective under experimental conditions, it remains unclear, if the effect is clinically apparent. The pro-aggregatory effect of etamsylate is based on the induction of an increase in P-selectin membrane expression on thrombocytes (7). P-selectin is an adhesion molecule that reduces the mobility of platelets and leukocytes on activated vascular endothelium (27). This effect of etamsylate appears to be dependent on the presence of vascular endothelium, since in one *in vitro* study, without contact with a damaged rabbit aorta segment, there was no formation of aggregates even though there was an increased release of P-selectin and activation of the platelets (28). Besides enhancing platelet aggregation and adhesion, the procoagulant effect of etamsylate involves vasoconstriction through prostacyclin inhibition (5). As an *in vitro* test, thromboelastography does not consider this influence of etamsylate on the vascular endothelium and vasoconstriction. Given the requirement for a damaged vascular wall segment, an *in vivo* test such as mucosal bleeding time, might better reflect the efficacy of etamsylate. However, a study in horses did not demonstrate significant changes in the template bleeding time after administration of 12.5 mg/kg etamsylate (29). Another study showed a significant effect on the mucosal bleeding time in rats when etamsylate was applied topically (11). When considering the use of these *in vivo* tests to demonstrate the effect of etamsylate, it must be noted that these tests are crude and subjective, with poor repeatability and a high intra-observer and inter-observer variability. The mucosal bleeding time can vary by as much as 80 s in an individual dog, even when performed by a single observer (30). Nevertheless, inclusion of additional platelet function testing (e.g., mucosal bleeding time) beyond thromboelastography might have been advantageous to further assess potential effects of etamsylate on primary hemostasis, particularly with respect to platelet adhesion and endothelial interactions.

Thromboelastographic results are also influenced by multiple pre-analytical and analytical variables, which could also have influenced the results in this study (25). Factors such as sampling site, sampling technique (31, 32), storage time, and resting temperature (33–35) are known to influence thromboelastography outcomes. However, in the present study, the results of two measurements taken at different time points were very similar, suggesting that thromboelastography is a reliable measurement method when performed correctly and in accordance to the standardized guidelines (25).

It is also possible that the selected dosage or the timing of post-administration sampling was suboptimal to demonstrate the pro-aggregatory effect. The assumed positive effect on primary hemostasis is primarily based on experimental *in vitro* studies, and transferability of these effects is debatable. In clinical studies, the demonstrated effect of etamsylate was not consistent (1). In horses, intravenously administration of 12.5 mg/kg etamsylate followed by blood collection at one and two hours did not result in significant differences in these parameters compared to the baseline values (36). On the contrary, when etamsylate was added to the blood samples *in vitro,* it caused changes indicating increased platelet activation and platelet aggregation (8, 37). However, an *in vitro* study using dog blood showed no influence of etamsylate (250 mmoL/L) on the parameters of thromboelastography, which depict primary blood coagulation (21). These differing results and the greater likelihood of detecting effects in experimental *in vitro* settings, could be attributed to the fact that higher concentrations of etamsylate are achieved in experimental *in vitro* studies as can be achieved in plasma in clinical studies. This raises the question of whether a higher clinical dose might result in a more pronounced effect. Especially since many studies reported a dose-dependent effect (11, 21) and doses of up to 200 mg/kg of etamsylate did not result in significant adverse events in dogs and cats (3). A limitation is that this study was conducted many years ago, and therefore the generalizability of using such a high dosage may be limited.

Both dosage and timing are critical when attempting to detect a procoagulant effect of etamsylate. Even though a study (24) showed the maximal effect after 60 min, it is possible that the effect can only be documented by analyzing samples at different time points or after a longer period of administration of etamsylate. As in studies showing an effect of etamsylate, the drug was administered orally four times daily and blood samples were collected on the fourth day (8).

After treatment with etamsylate the platelet count declined by approximately 10%, with an absolute mean difference of 20 × 10^9^/l between measurements before and after treatment. Also, prothrombin time increased by 1.1 s (median) after etamsylate administration. After Holm–Bonferroni step-down procedure, these changes were no longer significant. Notably, these changes were only observed in the subgroups without changes in coagulation parameters, including patients with platelet count > 80 × 10^9^/l or MA within reference range or patients which got etamsylate prophylactically. An increased aggregation of platelets and thrombus formation could reduce the number of circulating platelets (38) and might thus indicate a pro-aggregatory effect of etamsylate consistent with findings from previous experimental studies. However, a pro-aggregatory effect of etamsylate would be expected to increase MA, which was not observed in the present study. Overall, these changes were not considered clinically relevant.

As etamsylate is considered to act within primary hemostasis independently of platelet count, which is not assessed by the parameters mentioned above, no effect on PT, aPTT, platelet count, and fibrinogen was expected. Also, previous studies in humans and pigs did not observe any impact of etamsylate on these parameters (8, 13, 14).

No adverse events associated with etamsylate were observed during the study period. Previous studies in humans also did not find any significant adverse events (39–42).

The results of the study are limited by the heterogeneity of the study population. However, as etamsylate is approved for dogs with bleeding due to coagulopathy or trauma, and for prophylactic use, an effect in all study groups was suspected. In addition, each patient served as its own control, with all parameters compared to their respective baseline values before etamsylate administration. This approach minimized the impact of pre-existing coagulopathies on the results. Still, disease progression might have influenced the parameters and focusing exclusively on a single, more homogenous group (e.g., dogs with confirmed coagulopathies) would allow more targeted conclusion. This represents an important direction for future studies. Another limitation was the time of sample collection. The post etamsylate sample was collected 90 min after etamsylate administration, as a study (24) indicated the maximum effect 30–120 min after intravenously application. Detecting the clinical effect of etamsylate might, however, require additional or alternative sampling time points. Additionally, it should be mentioned that not all values of fibrinogen and K-value were available for every dog. This reduced sample size for these variables and may have decreased the statistical power to detect small differences. As these two variables are not primarily reflecting the suspected mechanism of action of etamsylate significant changes in these parameters were not suspected.

## 5 Conclusion

In this study, intravenous administration of etamsylate at a dose of 12.5 mg/kg in dogs was not associated with detectable changes in thromboelastographic parameters, clotting times, platelet count, or fibrinogen when assessed 60 min after administration. Therefore, at the investigated dose and time point, no effects on these hemostatic variables were observed. Possible effects at different doses, time points, or after repeated administration cannot be excluded and were beyond the scope of the present study.

## Data Availability

The original contributions presented in the study are included in the article/Supplementary material, further inquiries can be directed to the corresponding author.
