# Supplementary material for: Influence of etamsylate on coagulation parameters in dogs
Source: Front Vet Sci. 2026 Mar 4;13:1734418. doi: 10.3389/fvets.2026.1734418 (PMC12997416; doi:10.3389/fvets.2026.1734418)
Supplement: Supplementary file 1 [file Data_Sheet_1.docx]

**Supplements:**

| **parameters (n)** | **reference range** | **before etamsylate** | | **after etamsylate** | | ***p*-values** |
| --- | --- | --- | --- | --- | --- | --- |
|  |  | **median** | **range** | **median** | **range** |  |
| **R (min)**  **(n = 10)** | 1.8 – 8.6 | 5.2 | 3.5 – 15.2 | 5.8 | 2.8 – 13.4 | 1.000 |
| **K (min)**  **(n = 5)** | 1.3 – 5.7 | 2.1 | 1.1 – 3.6 | 2.3 | 1.2 – 3.6 | 1.000 |
| **angle (deg)**  **(n = 9)** | 36.9 – 74.6 | 39.3 | 3.9 – 73.6 | 38.3 | 4.6 – 74 | 1.000 |
| **MA (mm)**  **(n = 9)** | 42.9 – 67.9 | 27.4 | 3.1 – 62.9 | 20.7 | 3.9 – 65.4 | 1.000 |
| **LY30 (%)**  **(n = 9)** | 0 | 0 | 0 – 0.1 | 0.15 | 0 – 12.3 | 1.000 |
| **G (dyn/cm^2^)**  **(n = 9)** | 5796.6 – 13,885.9 | 2,225 | 160 – 8,477.1 | 1,546 | 112.5 – 9,450.9 | 1.000 |
| **platelet count  (x 10^9^/l)**  **(n = 10)** | 148 – 484 | 39 | 1 – 60 | 31.5 | 2 – 73 | 1.000 |
| **fibrinogen (mg/dl)**  **(n = 7)** | 88 – 560 | 155 | 112 – 943 | 181 | 91 – 464 | 1.000 |
| **PT (sec)**  **(n = 10)** | 13.8 – 23.2 | 22.7 | 13.5 – 41.4 | 23.8 | 12.3 – 45.8 | 1.000 |
| **aPTT (sec)**  **(n = 10)** | 10 – 13.1 | 12.6 | 9.7 – 51.3 | 13.4 | 11.5 – 41.1 | 1.000 |

Table S1: Coagulation times, platelet count, fibrinogen, and thromboelastography values of patients with platelet count below 80 x 10^9^/l (n = 10) before and after treatment with etamsylate.

aPTT = activated partial thromboplastin time, G = global clot strength (G = (5000xMA)/(100-MA)), K = k time, LY30 = clot lysis at 30 minutes, MA = maximal amplitude, PT = prothrombin time, R = reaction time. *P-*values ≤ 0.05 were considered significant.

Table S2: Coagulation times, platelet count, fibrinogen, and thromboelastography values of patients with platelet count above 80 x 10^9^/l (n = 20) before and after treatment with etamsylate.

| **parameters (n)** | **reference range** | **before etamsylate** | | **after etamsylate** | | ***p*-values** |
| --- | --- | --- | --- | --- | --- | --- |
|  |  | **median** | **range** | **median** | **range** |  |
| **R (min)**  **(n = 20)** | 1.8 – 8.6 | 4.2 | 2.4 – 7.1 | 4.8 | 2.4 – 6.6 | 1.000 |
| **K (min)**  **(n = 19)** | 1.3 – 5.7 | 1.8 | 0.8 – 4.7 | 1.6 | 0.9 – 3.9 | 0.744 |
| **angle (deg)**  **(n = 20)** | 36.9 – 74.6 | 65.8 | 15.3 – 78.9 | 65.0 | 23.3 – 78.8 | 1.000 |
| **MA (mm)**  **(n = 20)** | 42.9 – 67.9 | 59.1 | 16 – 81.6 | 60.3 | 17.6 – 80.9 | 1.000 |
| **LY30 (%)**  **(n = 20)** | 0 | 0 | 0 – 2 | 0 | 0 – 7.5 | 1.000 |
| **G (dyn/cm^2^)**  **(n = 20)** | 5,796.6 – 13,885.9 | 7,225 | 952.4 – 22,173.9 | 7,602 | 1,068.0 – 21,178.0 | 1.000 |
| **platelet count  (x 10^9^/l)**  **(n = 20)** | 148 – 484 | 231.5 | 108 – 442 | 225.5 | 101 – 391 | 0.080 |
| **fibrinogen (mg/dl)**  **(n = 15)** | 88 – 560 | 216 | 60 – 790 | 218 | 65.5 – 734 | 0.875 |
| **PT (sec)**  **(n = 19)** | 13.8 – 23.2 | 18.3 | 14.5 – 34 | 19.4 | 14.2 – 36 | 0.108 |
| **aPTT (sec)**  **(n = 19)** | 10 – 13.1 | 12.9 | 9.2 – 23 | 12.9 | 11.3 – 24.5 | 1.000 |

aPTT = activated partial thromboplastin time, G = global clot strength (G = (5000xMA)/(100-MA)), K = k time, LY30 = clot lysis at 30 minutes, MA = maximal amplitude, PT = prothrombin time, R = reaction time. *P-*values ≤ 0.05 were considered significant.

Table S3: Coagulation times, platelet count, fibrinogen, and thromboelastography values of patients with normal maximal amplitude values (n = 23) before and after treatment with etamsylate.

| **parameters (n)** | **reference range** | **before etamsylate** | | **after etamsylate** | | ***p*-values** |
| --- | --- | --- | --- | --- | --- | --- |
|  |  | **median** | **range** | **median** | **range** |  |
| **R (min)**  **(n = 23)** | 1.8 – 8.6 | 4.3 | 2.5 – 69.9 | 4.6 | 2.4 – 33.7 | 1.000 |
| **K (min)**  **(n = 23)** | 1.3 – 5.7 | 1.8 | 0.8 – 3.6 | 1.6 | 0.9 – 3.6 | 1.000 |
| **angle (deg)**  **(n = 23)** | 36.9 – 74.6 | 66.0 | 47.8 – 78.9 | 65.8 | 48.6 – 78.8 | 1.000 |
| **MA (mm)**  **(n = 23)** | 42.9 – 67.9 | 59.8 | 43.1 – 81.6 | 61.3 | 35.9 – 80.9 | 1.000 |
| **LY30 (%)**  **(n = 23)** | 0 | 0 | 0 – 2 | 0 | 0 – 7.5 | 1.000 |
| **G (dyn/cm^2^)**  **(n = 23)** | 5,796.6 – 13,885.9 | 7,225 | 3,787. 3 – 22,173.9 | 7,930 | 2,800.3 – 21,178.0 | 0.672 |
| **platelet count  (x 10^9^/l)**  **(n = 23)** | 148 – 484 | 227 | 23 – 319 | 199 | 9 – 254 | 0.140 |
| **fibrinogen (mg/dl)**  **(n = 19)** | 88 – 560 | 226 | 112 – 943 | 226 | 89 – 734 | 1.000 |
| **PT (sec)**  **(n = 22)** | 13.8 – 23.2 | 18.4 | 13.5 – 41.4 | 19.4 | 12.3 – 39.7 | 0.246 |
| **aPTT (sec)**  **(n = 22)** | 10 – 13.1 | 12.8 | 9.2 – 54.9 | 12.9 | 11.7 – 55.4 | 1.000 |

aPTT = activated partial thromboplastin time, G = global clot strength (G = (5000xMA)/(100-MA)), K = k time, LY30 = clot lysis at 30 minutes, MA = maximal amplitude, PT = prothrombin time, R = reaction time. *P-*values ≤ 0.05 were considered significant.

Table S4: Coagulation times, platelet count, fibrinogen, and thromboelastography values of patients with decreased maximal amplitude values (n = 6) before and after treatment with etamsylate.

| **parameters (n)** | **reference range** | **before etamsylate** | | **after etamsylate** | | ***p*-values** |
| --- | --- | --- | --- | --- | --- | --- |
|  |  | **median** | **range** | **median** | **range** |  |
| **R (min)**  **(n = 6)** | 1.8 – 8.6 | 7.15 | 2.4 – 15.2 | 7.25 | 4.3 – 13.4 | 1.000 |
| **K (min)**  **(n = 1)** | 1.3 – 5.7 | 4.7 | - | 3.9 | - | - |
| **angle (deg)**  **(n = 6)** | 36.9 – 74.6 | 13.8 | 3.9 – 49.4 | 17.6 | 4.6 – 48 | 1.000 |
| **MA (mm)**  **(n = 6)** | 42.9 – 67.9 | 8.5 | 3.1 – 41.2 | 5.5 | 3.9 – 39.6 | 1.000 |
| **LY30 (%)**  **(n = 6)** | 0 | 0 | 0 – 0.3 | 0.15 | 0 – 12.3 | 1.000 |
| **G (dyn/cm^2^)**  **(n = 6)** | 5,796.6 – 13,885.9 | 4,843 | 160.0 – 3,503.4 | 4,980 | 202.9 – 3,278.1 | 1.000 |
| **platelet count  (x 10^9^/l)**  **(n = 6)** | 148 – 484 | 45.8 | 1 – 141 | 45 | 2 – 109 | 1.000 |
| **fibrinogen (mg/dl)**  **(n = 3)** | 88 – 560 | 141 | 60 – 155 | 139 | 65.5 – 181 | 1.000 |
| **PT (sec)**  **(n = 6)** | 13.8 – 23.2 | 23.1 | 17.3 – 41.0 | 24.4 | 19.5 – 45.8 | 1.000 |
| **aPTT (sec)**  **(n = 6)** | 10 – 13.1 | 20.6 | 9.7 – 41.3 | 21.2 | 11.5 – 41.1 | 1.000 |

aPTT = activated partial thromboplastin time, G = global clot strength (G = (5000xMA)/(100-MA)), K = k time, LY30 = clot lysis at 30 minutes, MA = maximal amplitude, PT = prothrombin time, R = reaction time. *P-*values ≤ 0.05 were considered significant.

Table S5: Coagulation times, platelet count, fibrinogen, and thromboelastography values of patients with bleedings (n = 20) before and after treatment with etamsylate.

| **parameters (n)** | **reference range** | **before etamsylate** | | **after etamsylate** | | ***p*-values** |
| --- | --- | --- | --- | --- | --- | --- |
|  |  | **median** | **range** | **median** | **range** |  |
| **R (min)**  **(n = 20)** | 1.8 – 8.6 | 4.4 | 2.4 – 69.9 | 4.7 | 2.4 – 33.7 | 0.940 |
| **K (min)**  **(n = 14)** | 1.3 – 5.7 | 1.8 | 0.8 – 4.7 | 1.7 | 1.0 – 3.9 | 1.000 |
| **angle (deg)**  **(n = 19)** | 36.9 – 74.6 | 61.8 | 3.9 – 78.1 | 64.2 | 4.6 – 76.9 | 1.000 |
| **MA (mm)**  **(n = 19)** | 42.9 – 67.9 | 49.2 | 3.1 – 72.8 | 49.4 | 3.9 – 67.8 | 1.000 |
| **LY30 (%)**  **(n = 19)** | 0 | 0 | 0 – 0.6 | 0 | 0 – 12.3 | 0.580 |
| **G (dyn/cm^2^)**  **(n = 19)** | 5,796.6 – 13,885.9 | 4,843 | 160.0 – 13,382.4 | 4,980 | 202.9 – 10,528.0 | 1.000 |
| **platelet count  (x 10^9^/l)**  **(n = 20)** | 148 – 484 | 108 | 1 – 319 | 101 | 2 – 254 | 0.955 |
| **fibrinogen (mg/dl)**  **(n = 13)** | 88 – 560 | 216 | 60 – 943 | 196 | 65.5 – 464 | 0.984 |
| **PT (sec)**  **(n = 19)** | 13.8 – 23.2 | 21.8 | 13.5 – 41.4 | 22.3 | 12.3 – 45.8 | 0.960 |
| **aPTT (sec)**  **(n = 19)** | 10 – 13.1 | 14.6 | 9.7 – 54.9 | 14.1 | 11.5 – 55.4 | 1.000 |

aPTT = activated partial thromboplastin time, G = global clot strength (G = (5000xMA)/(100-MA)), K = kappa value, LY30 = clot lysis at 30 minutes, MA = maximal amplitude, PT = prothrombin time, R = reaction time. *P-*values ≤ 0.05 were considered significant.

Table S6: Coagulation times, platelet count, fibrinogen, and thromboelastography values of patients, that got etamsylate prophylactically prior to interventions with high bleeding risk (n = 10), before and after treatment with etamsylate.

| **parameters (n)** | **reference range** | **before etamsylate** | | **after etamsylate** | | ***p*-values** |
| --- | --- | --- | --- | --- | --- | --- |
|  |  | **median** | **range** | **median** | **range** |  |
| **R (min)**  **(n = 10)** | 1.8 – 8.6 | 4.4 | 3.6 – 5.9 | 4.8 | 2.9 – 6.2 | 1.000 |
| **K (min)**  **(n = 10)** | 1.3 – 5.7 | 1.8 | 0.8 – 3.0 | 1.6 | 0.9 – 2.5 | 1.000 |
| **angle (deg)**  **(n = 10)** | 36.9 – 74.6 | 65.8 | 50.8 – 78.9 | 65.0 | 56.7 – 78.8 | 1.000 |
| **MA (mm)**  **(n = 10)** | 42.9 – 67.9 | 59.0 | 53.4 – 81.6 | 63.7 | 52.6 – 80.9 | 1.000 |
| **LY30 (%)**  **(n = 10)** | 0 | 0 | 0 – 1.0 | 0 | 0 – 0.8 | 1.000 |
| **G (dyn/cm^2^)**  **(n = 10)** | 5,796.6 – 13,885.9 | 7,180 | 5,729.6 – 22,173.9 | 8,756 | 5,548.5 – 21,178.0 | 1.000 |
| **platelet count  (x 10^9^/l)**  **(n = 10)** | 148 – 484 | 274 | 214 – 442 | 252 | 156 – 391 | 0.322 |
| **fibrinogen (mg/dl)**  **(n = 9)** | 88 – 560 | 210 | 134 – 790 | 218 | 89 – 734 | 1.000 |
| **PT (sec)**  **(n = 10)** | 13.8 – 23.2 | 16.8 | 14.5 – 23.8 | 18.2 | 14.2 – 24.6 | 0.531 |
| **aPTT (sec)**  **(n = 10)** | 10 – 13.1 | 12.2 | 9.2 – 15.5 | 12.7 | 11.3 – 15.3 | 1.000 |

aPTT = activated partial thromboplastin time, G = global clot strength (G = (5000xMA)/(100-MA)), K = k time, LY30 = clot lysis at 30 minutes, MA = maximal amplitude, PT = prothrombin time, R = reaction time. *P-*values ≤ 0.05 were considered significant.
